# Meat, vegetables and genetic polymorphisms and the risk of colorectal carcinomas and adenomas

**DOI:** 10.1186/1471-2407-7-228

**Published:** 2007-12-19

**Authors:** Camilla F Skjelbred, Mona Sæbø, Anette Hjartåker, Tom Grotmol, Inger-Lise Hansteen, Kjell M Tveit, Geir Hoff, Elin H Kure

**Affiliations:** 1 Department of Laboratory Medicine, Section of Medical Genetics, Telemark Hospital, N-3710 Skien, Norway; 2 Telemark University College, Faculty of Arts and Sciences, Department of Environmental and Health Studies, Hallvard Eikas plass, N-3800 Bø i Telemark, Norway; 3 The Cancer Registry of Norway, Institute of Population-based Cancer Research, N-0310 Oslo, Norway; 4 The Cancer Center, Ulleval University Hospital, 0407 Oslo, Norway; 5 Department of Pathology, Ulleval University Hospital, 0407 Oslo, Norway

## Abstract

**Background:**

The risk of sporadic colorectal cancer (CRC) is mainly associated with lifestyle factors, particularly dietary factors. Diets high in red meat and fat and low in fruit and vegetables are associated with an increased risk of CRC. The dietary effects may be modulated by genetic polymorphisms in biotransformation genes. In this study we aimed to evaluate the role of dietary factors in combination with genetic factors in the different stages of colorectal carcinogenesis in a Norwegian population.

**Methods:**

We used a case-control study design (234 carcinomas, 229 high-risk adenomas, 762 low-risk adenomas and 400 controls) to test the association between dietary factors (meat versus fruit, berries and vegetables) genetic polymorphisms in biotransformation genes (*GSTM1*, *GSTT1*, *GSTP1 *Ile^105^Val, *EPHX1 *Tyr^113^His and *EPHX1 *His^139^Arg), and risk of colorectal carcinomas and adenomas. Odds ratio (OR) and 95% confidence interval (95% CI) were estimated by binary logistic regression.

**Results:**

A higher ratio of total meat to total fruit, berry and vegetable intake was positively associated with both high and low-risk adenomas, with approximately twice the higher risk in the 2^nd ^quartile compared to the lowest quartile. For the high-risk adenomas this positive association was more obvious for the common allele (Tyr allele) of the *EPHX1 *codon 113 polymorphism. An association was also observed for the *EPHX1 *codon 113 polymorphism in the low-risk adenomas, although not as obvious.

**Conclusion:**

Although, the majority of the comparison groups are not significant, our results suggest an increased risk of colorectal adenomas in individuals for some of the higher ratios of total meat to total fruit, berry and vegetable intake. In addition the study supports the notion that the biotransformation enzymes GSTM1, GSTP1 and EPHX1 may modify the effect of dietary factors on the risk of developing colorectal carcinoma and adenoma.

## Background

Colorectal cancer (CRC) is one of the most prevalent diseases in the industrialized Western countries. In Norway, with one of the highest incidence rates in the world, approximately 3500 new cases of CRC are diagnosed every year [1].

Epidemiological studies have attributed more than 85% of CRCs to environmental factors [2,3], particularly dietary factors [4]. Diets high in red meat and fat and low in fruit and vegetables are associated with an increased risk of CRC [5,6]. Although not entirely consistent, results from case-control and cohort studies suggest a modest association between intake of red meat and risk of CRC [7-9]. The mechanism behind the specific risk-enhancing effect of red meat is not precisely known, but there is evidence suggesting that components of red meat may independently increase the risk of CRC [10]. It has also been suggested that any association between high meat consumption and CRC may be a result of deficiencies in other protective dietary factors, such as fruit and vegetables [11,12]. In contrast to earlier studies, more recent epidemiological studies have generally not supported a strong influence of dietary fiber or fruit and vegetables, although these have other health benefits [10]. This has led to the notion that it is the ratio between meat and fruit/vegetable intake that is important. A study by Kapiszewska [13] showed that both the ratio between vegetables and meat consumption, as well as the ratio between the amount of energy from vegetables and animal products, can be used successfully to evaluate the relation of dietary pattern to cancer risk.

It is widely known that humans differ in their susceptibility to cancer. Difference in carcinogen metabolism might explain the differences in cancer susceptibility especially as most cancers are influenced by environmental factors [14]. Dietary effects might be modulated by genetic polymorphisms in biotransformation genes [15]. In the biotransformation system there are several interesting genes including microsomal epoxide hydrolase (*EPHX1*) (phase I) and gluthatione S-transferases (GSTs) (phase II), all with known polymorphisms [16,17].

The *EPHX1 *gene has a dual function and catalyzes the hydrolysis of arene, alkene and aliphatic epoxides to less reactive and more water-soluble dihydrodiols, but activates some polycyclic aromatic hydrocarbons into a more carcinogenic form [18]. A tyrosine to histidine substitution in exon 3 (Tyr^113^His) of the *EPHX1 *gene decreases *in vitro *enzyme activity by 40%, whereas a histidine to arginine substitution in exon 4 (His^139^Arg) increases *in vitro *enzyme activity by 25% [19].

GSTs are responsible for the glutathione conjugation and detoxify a number of various reactive species [20]. Genetic polymorphisms that result in reduced or absent activity or expression of the GST enzyme are known for most GST isoforms [21]. Research has mostly focused on the *GSTM1 *and *GSTT1 *deletion polymorphisms ("null" genotypes) resulting in absence of the GST μ1 and GST μl enzymes [22], and the *GSTP1 *Ile^105^Val polymorphism resulting in a less active enzyme [23].

Several lines of evidence support the adenoma – carcinoma sequence as the major path for colorectal carcinogenesis. The present study aimed to investigate the role of dietary factors (meat and fruit/vegetables) in combination with genetic factors in the different stages of colorectal carcinogenesis in a Norwegian population.

## Methods

The KAM cohort (Kolorektal cancer, Arv og Miljø) is based on the screening group of the Norwegian Colorectal Cancer Prevention study (The NORCCAP study) in the county of Telemark [24], and a series of clinical CRC cases (mean age 67.3, SD 11.2) operated on at Telemark Hospital (Skien) and Ulleval University Hospital (Oslo). Those invited to participate in the NORCCAP study were 20,780 men and women, age 50–64 years old, drawn by randomization from the population registry in Oslo (urban) and the county of Telemark (mixed urban and rural). They were invited to have a flexible sigmoidoscopy screening examination with or without (1:1) an additional faecal occult blood test (FOBT). Seven hundred and seventy-seven individuals were excluded according to exclusion criteria. The overall attendance rate was 65%. The ID number for the NORCCAP study at ClinicalTrials.gov is I NCT00119912 [25]. The screened cases in the NORCCAP study selected for colonoscopy were invited to participate in the KAM study (1044 cases agreed). During a limited period of time, after the screening study of NORCCAP was well established, controls (polyp free by flexible sigmoidoscopy) were invited to participate in the KAM study (400 controls agreed). The KAM cohort is based on an ethnic homogenous group of Norwegian origin. Written consent was obtained from all the participants. The KAM study is approved by the Regional Ethics Committee and the Data Inspectorate.

The KAM biobank consists of blood and tissue samples from 234 CRC cases (16 identified in the NORCCAP screening group and 218 from hospitals), 1044 individuals identified with polyps in the large intestine (229 high-risk and 762 low-risk adenomas, 53 hyperplastic polyps) and 400 controls, defined as individuals with normal findings at flexible sigmoidoscopy screening. Adenoma cases and controls were all drawn from the NORCCAP study. All of the participants completed a questionnaire on demographics, health status, dietary and smoking habits, alcohol consumption, physical exercise and occupation. The questionnaire contained information on a family history of adenomas and carcinomas, and the cases and controls included had no known personal history of genetic predisposition.

Diet was assessed in cases and controls using an extensive diet and lifestyle questionnaire, modeled on a questionnaire developed and validated for an American breast cancer study [26-28]. It was designed in a food frequency manner, asking the participants to record their average consumption, during the last year, of a wide range of typically Norwegian food items by ticking off fixed frequency and portion size boxes. Nine frequency choices, ranging from "never/less than once per month" to "2 or more per day" were given, whereas portion size was recorded as small, medium or large. The medium portion size was defined in the questionnaire (e.g. units, grams, dl). A small portion was defined as half or less than half of the medium portion, and a large portion was defined as one and a half or more of the medium portion. All questionnaires were checked for mistakes and completeness, and the participants who had delivered an inadequate questionnaire were contacted by phone in order to improve the quality of the data.

The adenocarcinomas were collected prior to chemo- or radiotherapy treatment. Two specialists in histopathology examined the histology of the adenomas independently in order to determine the tumor stage as mild, moderate or severe dysplasia. They reached the same conclusion in all cases.

Diminished numbers of available cases in the KAM biobank and analyzed cases are due to loss of samples during preparation and/or lack of information from the questionnaires. The 53 hyperplastic polyps were not included in the present analysis. A high-risk adenoma was defined as an adenoma measuring = 10 mm in diameter and/or with villous components and/or showing severe dysplasia [24].

Genomic DNA was isolated from blood samples according to standard procedures [29] with minor modifications as previously described [30].

Polymorphisms of the *GSTM1 *(null), *GSTT1 *(null) and *GSTP1 *(Ile^105^Val) genes were analyzed simultaneously by multiplex PCR as described by Nedelcheva Kristensen *et al*.[31].

The *EPHX1 *His^139^Arg polymorphism (rs2234922) was analyzed by a PCR-RFLP (restriction fragment length polymorphism) method as described by Hasset *et al*.[19].

Genotype analysis of the *EPHX1 *Tyr^113^His polymorphism (rs1051740) was carried out using the TaqMan allelic discrimination assay on a Sequence Detection System ABI 7000 (Applied Biosystems), using the primers forward: 5'-TCT CCT ACT GGC GGA ATG AAT T-3', reverse: 5'-CTG GCT GGC GTT TTG CA-3' and the probes T-allele: 5'-VIC-TCT CAA CAG ATA CCC TCA-MGB-3', C-allele: 5'-6-FAM-TCA ACA GAC ACC CTC A-MGB-3'. The polymorphism was determined in a 12 μl reaction containing 1× MasterMix, 200 nM of each probe, 900 nM primers, and ca 50 ng of genomic DNA. Cycling conditions were as follows: 50°C for 2 min, 95°C for 10 min, and 45 cycles of 95°C for 15 s and 60°C for 1 min. Controls were included in each run, and 10% of the samples were retyped with identical results.

Eight summary variables were created from the individual dietary items in the food frequency questionnaire to describe the intake of red meat, processed meat, total meat, raw vegetables, boiled vegetables, fruit and berries, total fruit, berries and vegetables and the ratio between total meat and fruit, berry and vegetable intake. For each individual dietary factor, the frequency and portion size were multiplied to obtain the amount in grams per day. The summary variables were then divided into quartiles based on distribution of consumption among controls.

Differences in characteristics between the control group and all the case groups were assessed using the χ^2 ^test for categorical variables and the Mann-Whitney test for continuous variables. P-values < 0.05 were considered significant. Odds ratios (ORs) and 95% confidence intervals (CI) were calculated using binary logistic regression analysis to assess the relationship between the dietary factors, each polymorphism and colorectal carcinoma or adenoma cases. Crude ORs were calculated, adjusted for age and gender only. In addition multiple logistic regression analyses were performed with an adjustment for age, gender, smoking dose, alcohol consumption, and when appropriate total meat consumption and total fruit, berry and vegetable consumption. In addition the modifying effect by genotype on dietary exposure (ratio of total meat to total fruit, berry and vegetable intake) was studied by stratification in a two by four table. All analyses were carried out using the statistical analysis software SPSS (Chicago, Illinois, USA) 12.0.1 for Windows.

## Results

Selected characteristics of cases and controls are shown in Table 1. The gender distribution was dissimilar in the control and the case groups. Cases were older and more often smokers. The low-risk adenoma cases had a higher alcohol intake than the controls. Low-risk adenoma cases had a higher total meat intake than controls, and the high and low-risk adenoma cases had a higher ratio of intake of total meat to total fruit, berries and vegetables.

**Table 1:** Distribution of age, gender, cigarette smoking, alcohol consumption and dietary factors among controls and cases with colorectal carcinomas and adenomas

	**Controls**	**Carcinomas**	**High-risk adenomas**	**Low-risk adenomas**
**No. of subjects**	400	234	229	762
				
**Gender^a^**				
Male, No (%)	157 (39.3)	128 (54.7)	151 (65.9)	456 (59.8)
Female, No (%)	243 (60.8)	106 (45.3)	78 (34.1)	306 (40.2)
				
**Age^b^**				
Mean, years (SD)	54.2 (3.3)	67.3(11.2)	57.3 (3.5)	57.3 (3.8)
				
**Smoking status**				
Never smoked, No (%)	156 (46.7)	67 (38.5)	52 (27.1)	206 (31.5)
Ever smoked, No (%)	178 (53.3)	107 (61.5)	140 (72.9)	448 (68.5)
Number of cigarette years^c^, median (25–75 percentile)	213 (58.8–362.0)	300 (120.0–546.0)	350 (210.3–484.8)	314 (137.0–468.0)
				
**Alcohol consumption**				
Never, No (%)	73 (21.8)	43 (25.3)	45 (22.6)	147 (21.9)
Ever, No (%)	262 (78.2)	127 (74.7)	154 (77.4)	523 (78.1)
Number of alcohol units per month^d^, median (25–75 percentile)	6.5 (2.5–16.0)	10.5 (4.0–25.0)	9.0 (5.0–20.1)	9.0 (4.5–19.0)
				
**Dietary intake in g/day, median (25–75 percentile)**				
**Red meat consumption**	25 (16.5–45.0)	24 (12.0–39.4)	27 (16.5–45.0)	27 (16.5–45.0)
**Processed meat consumption^e^**	70 (40.2–112.9)	62 (31.6–95.2)	77 (42.1–120.9)	77 (46.1–113.6)
**Total meat consumption^f^**	99 (63.5–154.5)	90 (53.4–134.5)	112 (71.0–163.0)	112 (74.9–154.9)
**Raw vegetable consumption**	52 (22.8–84.8)	41 (19.2–81.0)	42 (19.9–75.0)	45 (22.9–80.1)
**Boiled vegetable consumption^g^**	58 (34.9–94.7)	74 (39.2–108.1)	64 (38.7–100.3)	67 (40.2–108.3)
**Fruit and berry consumption^h^**	141 (74.1–224.3)	106 (51.9–190.1)	122 (54.7–200.5)	118 (59.7–199.6)
**Total fruit, berry and vegetable consumption**	265 (166.4–390.9)	250 (149.7–378.4)	230 (156.2–372.1)	241 (157.6–358.7)
**Ratio of total meat to total fruit, berry and vegetable intake^i^**	0.38 (0.19–0.71)	0.40 (0.20–0.63)	0.43 (0.26–0.76)	0.45 (0.27–0.82)

^a ^There are significant differences in the number of males and females between the control group and all the case groups, P < 10^-4^.

^b ^There are significant differences in age between the control group and all the case groups, P < 10^-4^.

^c ^There are significant differences in number of cigarette years (smoking dose) between the control group and all of the case groups, P = 0.001, P < 10^-4 ^and P < 10^-4^, respectively.

^d ^There is significant difference in number of alcohol units per month between the control group and the low-risk adenoma group, P = 0.013.

^e ^There is significant difference in processed meat consumption (g/day) between the control group and the low-risk adenoma group, P = 0.041.

^f ^There is significant difference in total meat consumption (g/day) between the control group and the low-risk adenoma group, P = 0.027.

^g ^There is significant difference in boiled vegetable consumption (g/day) between the control group and the low-risk adenoma group, P = 0.035.

^h ^There are significant differences in fruit and berry consumption (g/day) between the control group and the carcinoma and low-risk adenoma groups, P = 0.019 and P = 0.005, respectively.

^i ^There are significant differences in ratio of total meat to total fruit, berry and vegetable intake between the control group and the high- and low-risk adenoma groups, P = 0.035 and P = 0.003, respectively.

Missing values for cigarette smoking, alcohol consumption and dietary factors gave rise to diminished number of cases and controls.

The distribution of consumption of the dietary factors in quartiles is presented in Table 2, and the genotype distribution is shown in Table 3. The tables also present the estimates of ORs of colorectal carcinomas and adenomas associated with the various dietary factors and polymorphisms. The tables present only data from the multi-adjusted model which may be a better choice than the crude model in reducing potential confounding in the data.

**Table 2:** Association between intake of dietary factors and colorectal carcinomas and adenomas

	**Controls**	**Carcinomas**	**OR (95% CI)^a^**	**High-risk adenomas**	**OR (95% CI)^a^**	**Low-risk adenomas**	**OR (95% CI)^a^**
**Dietary factors^b^**							
**Total meat**							
≤ 64	72	37	1 (ref)	35	1 (ref)	107	1 (ref)
> 64 and ≤ 99	77	29	1.00 (0.43–2.32)	42	1.07 (0.56–2.06)	139	1.05 (0.66–1.67)
> 99 and ≤ 155	77	33	1.88 (0.82–4.31)	49	1.46 (0.76–2.78)	186	1.57 (1.00–2.46)
> 155	74	27	0.93 (0.38–2.30)	49	1.22 (0.63–2.37)	149	1.06 (0.66–1.70)
			P_trend _= 0.67		P_trend _= 0.41		P_trend _= 0.43
							
**Total fruit, berry and vegetable**							
≤ 166	76	36	1 (ref)	53	1 (ref)	159	1 (ref)
> 166 and ≤ 265	73	35	0.82 (0.36–1.88)	49	1.00 (0.55–1.80)	168	1.12 (0.73–1.74)
> 265 and ≤ 391	75	27	0.83 (0.36–1.92)	37	0.78 (0.41–1.46)	130	1.02 (0.65–1.59)
> 391	75	28	1.52 (0.67–3.45)	36	1.03 (0.55–1.95)	124	0.99 (0.63–1.57)
			P_trend _= 0.37		P_trend _= 0.86		P_trend _= 0.87
							
**Ratio of total meat to total fruit, berry and vegetable intake**							
≤ 0.19	75	27	1 (ref)	22	1 (ref)	90	1 (ref)
> 0.19 and ≤ 0.38	74	33	1.66 (0.72–3.84)	55	**2.59 **(1.31–5.14)	146	**1.73 **(1.08–2.75)
> 0.38 and ≤ 0.71	75	45	1.58 (0.69–3.63)	45	1.49 (0.73–3.02)	176	**1.68 **(1.06–2.67)
> 0.71	75	21	0.62 (0.23–1.62)	53	1.92 (0.95–3.91)	169	1.39 (0.85–2.25)
			P_trend _= 0.39		P_trend _= 0.37		P_trend _= 0.29

^a ^Adjusted for age, gender, smoking dose and alcohol consumption. In addition 'Total meat' and 'Total fruit, berry and vegetable' are mutually adjusted for when assessing their main effect. These variables were not adjusted for when the ratio between them was assessed.

^b ^Quantities are gram per day. Categories based on quartiles. Numbers vary between the dietary factors due to missing dietary data.

ref: served as reference category.

**Table 3:** Association between *GST *and *EPHX1 *genotypes and colorectal carcinomas and adenomas

	**Controls**	**Carcinomas**	**OR (95% CI)^a^**	**High-risk adenomas**	**OR (95% CI)^a^**	**Low-risk adenomas**	**OR (95% CI)^a^**
**Genotypes^b^**							
***GSTM1***							
Present	148	53	1 (ref)	84	1 (ref)	262	1 (ref)
Null	151	55	1.22 (0.65–2.30)	89	0.98 (0.63–1.52)	313	1.09 (0.80–1.49)
							
***GSTT1***							
Present	262	93	1 (ref)	143	1 (ref)	488	1 (ref)
Null	37	15	1.37 (0.57–3.31)	30	1.36 (0.72–2.55)	87	0.99 (0.63–1.58)
							
***GSTP1 *Ile^105^Val**							
Ile/Ile	119	51	1 (ref)	57	1 (ref)	240	1 (ref)
Ile/Val	140	50	0.82 (0.43–1.59)	90	1.27 (0.78–2.06)	272	1.00 (0.71–1.39)
Val/Val	40	7	0.38 (0.12–1.23)	26	1.29 (0.65–2.59)	63	0.79 (0.48–1.32)
Ile/Val + Val/Val	180	57	0.71 (0.38–1.32)	116	1.27 (0.80–2.02)	335	0.95 (0.69–1.31)
			P_trend _= 0.13		P_trend _= 0.35		P_trend _= 0.49
							
***EPHX1 *Tyr^113^His**							
Tyr/Tyr	132	52	1 (ref)	75	1 (ref)	304	1 (ref)
Tyr/His	134	41	0.74 (0.37–1.47)	81	0.90 (0.57–1.44)	229	**0.69 **(0.50–0.97)
His/His	33	9	1.04 (0.33–3.21)	17	0.87 (0.40–1.88)	43	**0.56 **(0.32–0.99)
Tyr/His + His/His	167	50	0.78 (0.41–1.51)	98	0.90 (0.57–1.40)	272	**0.67 **(0.49–0.92)
			P_trend _= 0.68		P_trend _= 0.63		P_trend _= 0.01
							
***EPHX1 *His^139^Arg**							
His/His	190	56	1 (ref)	106	1 (ref)	347	1 (ref)
His/Arg	90	27	1.24 (0.60–2.57)	59	1.37 (0.85–2.21)	207	1.24 (0.88–1.74)
Arg/Arg	19	18	2.70 (0.96–7.58)	8	0.62 (0.23–1.70)	22	0.51 (0.25–1.06)
His/Arg + Arg/Arg	109	45	1.52 (0.79–2.93)	67	1.22 (0.77–1.91)	229	1.11 (0.80–1.53)
			P_trend _= 0.09		P_trend _= 0.82		P_trend _= 0.83

^a ^Adjusted for age, gender, smoking dose, alcohol consumption, and the consumption of total meat and total fruit, berry and vegetable.

^b ^Numbers vary between analyzed genotypes due to missing samples and/or failed analysis.

ref: the genotype served as reference category.

No significant trends were observed in the data of Table 2. Total meat consumption showed a borderline significant association with low-risk adenomas in the 3^rd ^quartile, OR of 1.57 (95% CI 1.00–2.46). No significant results were observed for total fruit, berry and vegetable consumption. The ratio of total meat to total fruit, berry and vegetable intake was positively associated with both high and low-risk adenomas in the 2^nd ^quartile (and 3^rd ^quartile for low-risk adenomas) with approximately twice the higher risk compared to the lowest quartile (ORs of 2.59 (95% CI 1.31–5.14) and 1.73 (95% CI 1.08–2.75), respectively). We have also tested the dietary exposures as binary variables, but these results gave less information than the chosen model (data not shown).

The frequencies for the *GSTM1 *null allele, *GSTT1 *null allele, *GSTP1 *105Val allele, *EPHX1 *113His allele and *EPHX1 *139Arg allele among the controls were respectively, 0.52, 0.13, 0.37, 0.32 and 0.21 (Table 3). The genotype distributions were all in Hardy-Weinberg equilibrium and the distributions of the alleles are in agreement with those found in other Caucasian populations [32-34]. No significant associations were found between the *GST *polymorphisms and risk of colorectal carcinomas and adenomas. The *EPHX1 *113His allele was associated with a decreased risk in low-risk adenomas with OR of 0.67 (95% CI 0.49–0.92), P_trend _= 0.01. No significant associations were seen between the *EPHX1 *139Arg allele and carcinomas and adenomas. Since the strongest association seems to be with the Arg/Arg genotype we have compared this genotype with the combined His/His and His/Arg genotypes as a reference. The Arg/Arg genotype was associated with decreased risk of low-risk adenomas only, OR of 0.48 (95% CI 0.23–0.98) (data not shown). We have also tested the combined effect of the two polymorphisms in *EPHX1 *(i.e., Tyr^113^His and His^139^Arg). Predicted mEPHX activities were classified as normal, fast and slow [35]. The same tendency was observed as for the separate genotypes (data not shown).

Table 4 display the results from analyses of the effect of the ratio of total meat to total fruit, berry and vegetable intake on the risk of colorectal carcinomas and adenomas stratified by genotype. The case groups are stratified based on polymorphic status, in most common allele or any polymorphic allele. Only data from the multi-adjusted model is presented.

**Table 4:** Ratios of total meat to total fruit, berry and vegetable intake and the risk of colorectal carcinomas and adenomas by *GST *and *EPHX1 *genotypes

		**OR (95% CI)^a ^(N_cases_/N_controls_)**							
**Case group**	**Ratio of total meat to total fruit, berry and vegetable intake^b^**	***GSTM1***		***GSTP1 *Ile^105^Val**		***EPHX1 *Tyr^113^His**		***EPHX1 *His^139^Arg**	

		Present	Null	Ile/Ile	Ile/Val+Val/Val	Tyr/Tyr	Tyr/His+His/His	His/His	His/Arg+Arg/Arg

**Carcinomas**	**Q**_1_	1 (ref) (11/39)	1 (ref) (11/36)	1 (ref) (7/32)	1 (ref) (15/43)	1 (ref) (10/33)	1 (ref) (10/42)	1 (ref) (9/51)	1 (ref) (11/24)
	**Q**_2_	0.84 (0.20–3.47) (11/33)	2.99 (0.85–10.5) (18/40)	2.01 (0.53–7.67) (16/27)	1.52 (0.43–5.35) (13/46)	3.28 (0.63–17.0) (17/29)	1.65 (0.51–5.41) (12/44)	**4.67 **(1.21–18.0) (17/48)	0.69 (0.17–2.89) (12/25)
	**Q**_3_	1.05 (0.31–3.57) (19/43)	2.39 (0.63–9.02) (18/32)	1.85 (0.48–7.18) (15/31)	1.22 (0.36–4.13) (22/44)	2.59 (0.47–14.3) (18/36)	1.34 (0.40–4.42) (17/39)	2.78 (0.71–11.0) (20/45)	0.63 (0.16–2.55) (14/30)
	**Q**_4_	0.49 (0.10–2.41) (12/33)	0.78 (0.19–3.23) (8/42)	1.61 (0.41–6.40) (13/28)	**0.11 **(0.01–0.88) (7/47)	0.70 (0.11–4.59) (7/33)	0.63 (0.16–2.52) (11/42)	0.90 (0.20–4.13) (10/46)	0.34 (0.07–1.72) (8/29)
		P_trend _= 0.55	P_trend _= 0.51	P_trend _= 0.58	P_trend _= 0.10	P_trend _= 0.56	P_trend _= 0.51	P_trend _= 0.74	P_trend _= 0.21
									
**High-risk Adenomas**	**Q**_1_	1 (ref) (11/39)	1 (ref) (11/36)	1 (ref) (9/32)	1 (ref) (13/43)	1 (ref) (5/33)	1 (ref) (17/42)	1 (ref) (15/51)	1 (ref) (7/24)
	**Q**_2_	**3.52 **(1.26–9.85) (32/33)	2.05 (0.80–5.26) (23/40)	2.54 (0.85–7.54) (20/27)	**2.67 **(1.09–6.51) (35/46)	**8.98 **(2.54–31.8) (33/29)	1.26 (0.52–3.05) (22/44)	**2.55 **(1.05–6.24) (29/48)	2.68 (0.88–8.19) (26/25)
	**Q**_3_	1.49 (0.53–4.21) (22/43)	1.57 (0.59–4.20) (23/32)	1.32 (0.42–4.16) (12/31)	1.42 (0.57–3.56) (33/44)	3.38 (0.90–12.75) (16/36)	1.08(0.45–2.63) (29/39)	2.00 (0.81–4.94) (31/45)	0.93 (0.28–3.10) (14/30)
	**Q**_4_	1.83 (0.59–5.63) (19/33)	1.91 (0.74–4.92) (32/42)	1.89 (0.60–5.95) (16/28)	1.68 (0.67–4.19) (35/47)	3.65 (1.00–13.3) (21/33)	1.44 (0.59–3.52) (30/42)	1.94 (0.77–4.88) (31/46)	1.65 (0.52–5.26) (20/29)
		P_trend _= 0.81	P_trend _= 0.34	P_trend _= 0.59	P_trend _= 0.74	P_trend _= 0.67	P_trend _= 0.51	P_trend _= 0.33	P_trend _= 0.94
									
**Low-risk adenomas**	**Q**_1_	1 (ref) (36/39)	1 (ref) (54/36)	1 (ref) (44/32)	1 (ref) (46/43)	1 (ref) (38/33)	1 (ref) (52/42)	1 (ref) (57/51)	1 (ref) (33/24)
	**Q**_2_	**2.62 **(1.28–5.35) (65/33)	1.32 (0.70–2.48) (77/40)	1.38 (0.67–2.83) (52/27)	**2.02 **(1.08–3.77) (90/46)	**2.65 **(1.28–5.46) (83/29)	1.15 (0.61–2.14) (59/44)	1.67 (0.94–2.98) (82/48)	1.86 (0.83–4.15) (60/25)
	**Q**_3_	1.89 (0.96–3.74) (81/43)	1.63 (0.85–3.12) (94/32)	1.42 (0.71–2.85) (70/31)	1.86 (1.00–3.47) (105/44)	**2.07 **(1.02–4.20) (93/36)	1.36 (0.73–2.54) (82/39)	1.64 (0.91–2.93) (97/45)	1.70 (0.78–3.71) (78/30)
	**Q**_4_	1.89 (0.90–3.98) (80/33)	1.06 (0.55–2.04) (88/42)	1.25 (0.60–2.65) (74/28)	1.43 (0.75–2.73) (94/47)	1.57 (0.74–3.35) (90/33)	1.23 (0.65–2.33) (79/42)	1.52 (0.83–2.79) (111/46)	1.13 (0.50–2.56) (58/29)
		P_trend _= 0.25	P_trend _= 0.80	P_trend _= 0.55	P_trend _= 0.48	P_trend _= 0.49	P_trend _= 0.46	P_trend _= 0.22	P_trend _= 0.98

^a ^OR adjusted for age, gender, smoking dose and alcohol consumption.

^b ^Quartiles of ratio of total meat to total fruit, berry and vegetable intake, per day: ≤ 0.19, >0.19 and ≤ 0.38, > 0.38 and ≤ 0.71, > 0.71.

ref: The combination served as a reference.

No significant trends were observed. For carcinomas we did not find any significant association with ratio of total meat to total fruit, berry and vegetable intake, and the combination with genotype gives few significant results. A reduced risk (Q_4_) was observed in individuals with the *GSTP1 *codon 105 variant allele (Val allele). In addition a positive association between ratio (Q_2_) and CRC risk was more obvious in individuals with the *EPHX1 *codon 139 common allele (His allele).

A higher ratio of total meat to total fruit, berry and vegetable intake was positively associated with colorectal adenomas. For the high-risk adenomas this positive association was more obvious for the common allele (Tyr allele) of the *EPHX1 *codon 113 polymorphism. The same was seen for the *EPHX1 *codon 113 polymorphism in the low-risk adenomas, although not so obvious. The ratio (Q_2_) was also associated with an increased risk of high-risk and low-risk adenomas in individuals with the *GSTM1 *common allele and the *GSTP1 *codon 105 variant allele (Val allele).

Statistical analysis of the modifying effect by the *GSTT1 *polymorphism on the ratio of total meat to total fruit, berries and vegetables intake was not possible to perform due to low sample size.

## Discussion

Sporadic CRC is a consequence of multiple risk factors, among which the interaction between environmental and genetic factors is of particular interest. In this case-control study we investigated whether the consumption of meat and fruit, berries and vegetables or selected genotypes was associated with risk of developing colorectal carcinomas and adenomas alone or if the genotypes could modify the results.

We found only a weak positive association between total meat consumption and low-risk adenomas. Lack of significant association with the cancer group and the high-risk adenoma group, may be due to small sample size. There appears to be consistent evidence from epidemiological data that intake of red meat is positively related to risk of developing CRC [8,9]. Enhanced risk of colorectal adenomas, an established precursor lesion of CRC, has also been associated with high levels of red meat consumption in some studies [36,37] but not in others [38,39].

We did not detect any association between fruit, berry and vegetable intake and risk of developing colorectal carcinomas or adenomas. The association between fruit and vegetables consumption has not been consistently proven in other studies. The majority of case-control studies show an inverse association between intake of vegetables and colon cancer risk, while prospective studies show no association [40]. Distribution of meat, vegetable, fruit and berry consumption in our study is in agreement with the NORKOST survey, a nation-wide survey of a representative sample of the adult population in Norway [41].

The ratio between total meat and total fruit, berry and vegetable intake yielded an increased risk of high and low-risk adenomas for the 2^nd ^quartile (and also the 3^rd ^quartile for low-risk adenomas) compared to the lower quartile. This result may apply to the general Norwegian population which has little variation in intake of meat, fruit, berries and vegetables. The result is in agreement with the result of Turner *et al*.[42] who showed that subjects who consumed small amounts of fruit and vegetables per month and many servings of red meat were at greater risk of CRC. The results also support the hypothesis of Kapiszewska [13] who has argued that it is not the consumption of a single food product or an individual component of diet, but rather a proper ratio of vegetables to meat consumption that contributes to cancer prevention. Lack of balance between the amount of meat and fruit, berries and vegetables may lead to the accumulation of damaged DNA, initiating DNA instability and inducing cancer development.

The *GSTM1*, *GSTT1 *and *GSTP1 *polymorphisms were not associated with risk of colorectal carcinomas and adenomas in this study. This is consistent with the results in some studies [33,43], but only partly in others [44].

The *EPHX1 *113His allele was associated with a decreased risk of low-risk adenomas. The *EPHX1 *codon 139 polymorphism was not associated with risk of carcinomas and adenomas in this study. Contradictory data have been provided by different studies investigating the *EPHX1 *codon 113 and *EPHX1 *codon 139 polymorphisms in relation to the risk of colorectal carcinomas and adenomas [45-50].

There is conflicting evidence regarding the role of *GST *and *EPHX1 *polymorphisms in CRC susceptibility. Since the enzymes have detoxifying activity, it would be expected that, rather than affecting the risk of cancer per se, they would modify risk when exposed to potential carcinogens [22].

To evaluate if the genetic polymorphisms in *GST *and *EPHX1 *modify colorectal carcinoma and adenoma risk in relation to ratio of total meat to total fruit, berry and vegetable intake, we stratified the case groups based on the most common allele or any polymorphic allele.

The most convincing evidence of a modifying effect by genotype on dietary exposure was found between *EPHX1 *codon 113 in adenomas and *EPHX1 *codon 139 in carcinomas. In our study, for both the *EPHX1 *codon 113 and *EPHX1 *codon 139 polymorphisms, the increased risk associated with a higher ratio of total meat to total fruit, berries and vegetables was confined to individuals homozygous for the most common alleles (*EPHX1 *113Tyr allele and *EPHX1 *139His allele). To our knowledge no studies have been published on the association between *GST *and *EPHX1 *polymorphisms and the ratio of total meat to total fruit, berry and vegetable intake, but some studies have looked at the gene-diet interaction with meat and fruit/vegetables separately. There are few reports on the association between *EPHX1 *polymorphisms and dietary factors. Cortessis *et al*.[46] reported increased risk of adenomas in individuals who ate their meat well done and had a high predicted *EPHX1 *activity (combination with 3 or 4 of the more stable alleles, defined as the Y (tyrosine) variant at exon 3 and the R (arginine) variant at exon 4), whereas Ulrich *et al*.[51] reported elevated adenoma risks associated with low predicted activity in those with high intake of cooked meat. Other reports on *EPHX1 *and CRC and adenomas did not find evidence for an interaction with meat intake [42,47,48,50]. One report has suggested a weak interaction between fruit consumption and the *EPHX1 *113Tyr allele (common allele) [42], but due to unusual allele frequencies and unexpected pattern of results (greatest risk reduction in the intermediate genotype group) this finding must be interpreted with caution. The biochemical pathways in which EPHX1 participates involve numerous enzymes. The role of EPHX1 itself maybe quite complex, as the enzyme has broad substrate specificity and tissue distribution. Expression of EPHX1 is both induced and inhibited by exogenous compounds [52]. While EPHX1 usually results in detoxification of xenobiotic substances, it is also involved in the metabolic activation of polycyclic aromatic hydrocarbons found in cooked meat, triggering the formation of highly reactive metabolites that can damage DNA, RNA and protein components [35,53]. EPHX1 activity may also be modulated by components in fruit and vegetables. It is likely that EPHX1 could modify the association between diet and colorectal carcinoma and adenoma risk.

In addition we found weak evidence of a modifying effect by the *GSTP1 *and *GSTM1 *genotypes on the ratio of total meat to total fruit, berry and vegetable intake in adenomas. The increased risk associated with a higher ratio of total meat to total fruit, berries and vegetables is restricted to individuals with the *GSTP1 *codon 105 variant allele (Val allele), and to the *GSTM1 *common allele. Since there are no significant trends and only a few significant single results caution is required in interpretation of our findings.

Studies have shown that higher levels of DNA damage are associated with the *GSTM1/T1 *null genotypes and the *GSTP1 *105Val allele [54,55]. The higher adenoma risk with a low capacity *GST *variant could directly reflect the slower processing of genotoxic compounds associated with meat intake. In addition a high ratio of meat to fruit, berries and vegetables may increase the risk of carcinogenesis.

Isothiocyanates (component of fruit and vegetables) are substrates for GSTs [56-58] and thus GSTs contribute to the excretion of isothiocyanates. The involvement of GSTs in isothiocyanate metabolism has led to the hypothesis that, through slower excretion of isothiocyanates from the body in individuals with genetic variants associated with lower GST capacity, isothiocyanates have greater opportunity to exert their chemo-protective effects in these individuals [59].

As with any case-control study, there is a possibility of bias in recall of diet leading to spurious associations. But for the assessment of gene-environment interactions this is not an issue, since there is no corresponding risk of bias in relation to genotype. We cannot exclude the possibility that the associations may be confounded or modified by other genetic or dietary factors. The CRC cases and controls have not been matched by age, which may affect the results in our study. However, the controls were recruited from the same population as the adenoma and carcinoma cases. Further, our controls have been screened and found polyp free by flexible sigmoidoscopy and the risk of any of them having colon cancer at the time of inclusion is not very likely. It has been estimated that less than 3% of individuals with no adenoma at flexible sigmoidoscopy are likely to harbor an undiagnosed proximal advanced colorectal neoplasia [60].

Due to the high number of multiple tests and comparisons in the present study, some statistically significant associations have undoubtedly occurred by chance, and caution is required in their interpretation. The results thus need to be verified by further studies with particular focus on the ratio between meat and fruit, berry and vegetable intake and the possible modifying effects of biotransformation genes.

## Conclusion

Although, the majority of the comparison groups are not significant, our results suggest an increased risk of colorectal adenomas in individuals for some of the higher ratio of total meat to total fruit, berry and vegetable intake. An unbalanced consumption of meat and fruit, berries and vegetables may lead to accumulation of DNA damage and thus increased risk of cancer development. In addition the study supports the notion that the biotransformation enzymes GSTM1, GSTP1 and EPHX1 may modify the effect of dietary factors on the risk of developing colorectal carcinoma and adenoma.

## Competing interests

The author(s) declare that they have no competing interests.

## Authors' contributions

CFS prepared the first draft of the paper. She prepared, with contribution from MS, the dietary data for statistical analysis. She did the data analysis and participated in genotyping of the samples. MS also contributed to the manuscript and with advice in the data analysis, and she participated in genotyping of the samples. AH advised on handling of the dietary data and statistical analysis. GH together with TG and KMT were responsible for designing and administering the NORCCAP clinical trial. In addition GH and TG provided advice on the statistical analysis. ILH participated in collection and quality control of the questionnaires. EHK initiated the KAM study and also organized it. She contributed with advice on data analysis and was responsible for the revisions of the manuscript. All authors discussed the results, contributed to interpretation of the results and the final manuscript.

## Pre-publication history

The pre-publication history for this paper can be accessed here:


